# Synthesis, Antibacterial Evaluation and QSAR of α-Substituted-*N_4_*-Acetamides of Ciprofloxacin and Norfloxacin

**DOI:** 10.3390/antibiotics3030244

**Published:** 2014-06-25

**Authors:** Amjad M. Qandil, Lorca O. Al-Zoubi, Amal G. Al-Bakri, Haneen A. Amawi, Qosay A. Al-Balas, Abdulmalik M. Alkatheri, Abdulkareem M. Albekairy

**Affiliations:** 1 Department of Medicinal Chemistry and Pharmacognosy, Faculty of Pharmacy, Jordan University of Science and Technology, P.O. Box (3030), Irbid 22110, Jordan; E-Mails: lolo_osama@hotmail.com (L.O.A.-Z.); qabalas@just.edu.jo (Q.A.A.-B.); 2 College of Pharmacy, King Saud bin Abdulaziz University for Health Sciences, Riyadh 11426, Saudi Arabia; E-Mails: katheria@ngha.med.sa (A.M.A.); bekairya@ngha.med.sa (A.M.A.); 3 Department of Pharmaceutics and Pharmaceutical Technology, Faculty of Pharmacy, University of Jordan, Amman 11942, Jordan; E-Mail: agbakri@ju.edu.jo; 4 Department of Clinical Pharmacy, Faculty of Pharmacy, Jordan University of Science and Technology, P.O. Box (3030), Irbid 22110, Jordan; E-Mail: haneen_pharma@yahoo.com; 5 King Abdulaziz Medical City, National Guard Health Affairs, Riyadh 11426, Saudi Arabia

**Keywords:** antibacterial activity, drug design, Gram-positive bacteria, QSAR, quinolones, synthesis

## Abstract

Twenty six α-substituted *N_4_*-acetamide derivatives of ciprofloxacin (CIPRO) and norfloxacin (NOR) were synthesized and assayed for antibacterial activity against *Pseudomonas aeruginosa*, *Escherichia coli*, *Staphylococcus aureus* and *Bacillus subtilis*. The derivatives were primarily more active against Gram-positive bacteria. The CIPRO derivatives, **CD-7** (Ar = 3-chlorophenyl), **CD-9** (Ar = 2-pyrimidyl) and **CD-10** (α-phenyl, Ar = 2-pyrimidyl), exhibited lower MIC values, 0.4–0.9 μM, against *Staphylococcus aureus* than CIPRO, while only compound **CD-10** exhibited better activity, 0.1 μM, against *Bacillus subtilis* than CIPRO. In addition, compounds **CD-5** (Ar = 2-methoxyphenyl), **CD-6** (α-phenyl, Ar = 2-methoxyphenyl), **CD-7** (Ar = 3-Chlorophenyl), **CD-8** (α-phenyl, Ar = 3-chlorophenyl) and **CD-9** (Ar = 2-pyrimidyl) showed MIC values below 1.0 μM against this strain. The NOR derivatives showed lower activity than NOR itself against *Staphylococcus aureus*, although **ND-6** (α-phenyl, Ar = 2-methoxyphenyl) and **ND-7** (Ar = 3-chlorophenyl) showed MIC values less than 2 μM. Two NOR derivatives, **ND-7** and **ND-6**, exhibited MIC values of 0.7 and 0.6, respectively, which were comparable to that of NOR against *Bacillus subtilis*, while compounds **ND-8** (α-phenyl, Ar = 3-chlorophenyl) and **ND-10** (α-phenyl, Ar = 2-pyrimidyl) exhibited MIC values less than 1.0 μM against the same strain. QSAR revealed that while polarity is the major contributing factor in the potency against *Staphylococcus aureus*, it is balanced by lipophilicity and electron density around the acetamide group. On the other hand, electron density around the introduced acetamide group is the major determining factor in the activity against *Bacillus subtilis*, with a lesser and variable effect for lipophilicity.

## 1. Introduction

**Figure 1:**
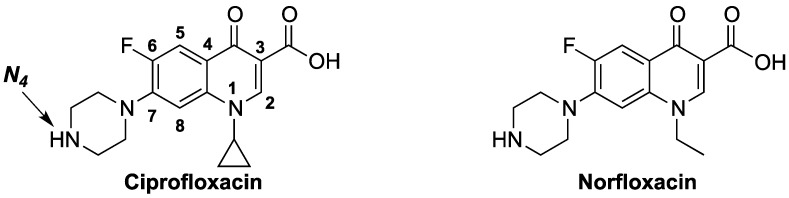
The chemical structures of ciprofloxacin (CIPRO) and norfloxacin (NOR).

Ciprofloxacin (CIPRO) and norfloxacin (NOR), Figure 1, are two simple and broad-spectrum fluoroquinolones. Fluoroquinolones are synthetic antibacterial agents that exhibit activity against, but not limited to, Enterobacteria, Mycobacteria, *Pseudomonas* spp., Streptococci (including pneumococci) and Staphylococci [1,2]. This group of compounds exert their antibacterial action by inhibiting bacterial *DNA gyrase* (*topoisomerase II*), which is the primary target in Gram-negative bacteria (e.g., *E. coli* and *Neisseria gonorrhoeae*), and *topoisomerase IV*, which is their primary site of action in Gram-positive bacteria (e.g., *S. aureus* and *S. pneumonia*) [3]. Structural modifications of this ubiquitous class of antibacterial agents have afforded compounds with reduced adverse effects, enhanced potency and/or better efficacy in resistant bacterial strains [4]. One important site of modification is position-7 (C7) of the quinolone nucleus. The nature of the substituent at C7 can affect potency, the spectrum of activity, the pharmacokinetic proprieties and the side effects [5,6,7]. The most commonly introduced substituent at C7 is a heterocyclic amine, such as the piperazine ring found in CIPRO and NOR, Figure 1. The secondary amine in the piperazine ring is responsible, in part, for the pharmacokinetic profile of ciprofloxacin, and it is also blamed for its CNS side effects [8]. Recently, there have been reports of some interesting *N_4_*-substitution patterns that resulted in compounds with narrowed spectrum of activity. It has been argued recently that the use of antibacterial agents with a narrow spectrum of activity can reduce the infection with certain organisms and reduce the emergence of resistance [9,10,11]. This is particularly important, because the fast spread of resistant bacterial strains is a major challenge in treating bacterial infections [12,13].

**Figure 2:**
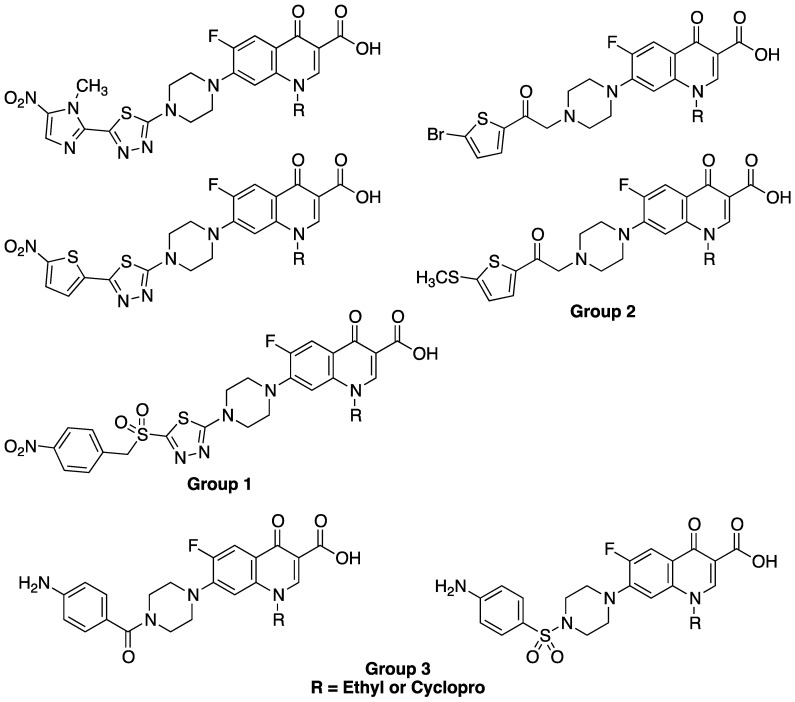
The chemical structures of some *N_4_*-substituted CIPRO and NOR derivatives with selective activity against Gram-positive bacteria.

Some examples of such *N_4_*-susbtituted CIPRO and NOR analogs with a narrow spectrum of activity are illustrated in Figure 2. Foroumadi *et al.* has reported the synthesis and antibacterial evaluation of a series of thiadiazolylpiperazine derivatives (**Group 1**, Figure 2) and thiophenylpiperazine derivatives (**Group 2**, Figure 2) of CIPRO and NOR and showed that they exhibited enhanced Gram-positive selectivity [14,15,16,17,18]. Other authors have reported that some *N*-phenylsulfonylpiperazinyl [19,20] and *N*-benzoylpiperazinyl [21] derivatives of CIPRO and NOR (**Group 3**, Figure 2) have also exhibited selectivity against Gram-positive bacteria. All of the above examples either contain a bulky aryl-containing substituent at the *N_4_* of the piperazine at C7 or an amide or a sulfonamide group that made *N_4_* non-basic.

**Figure 3:**
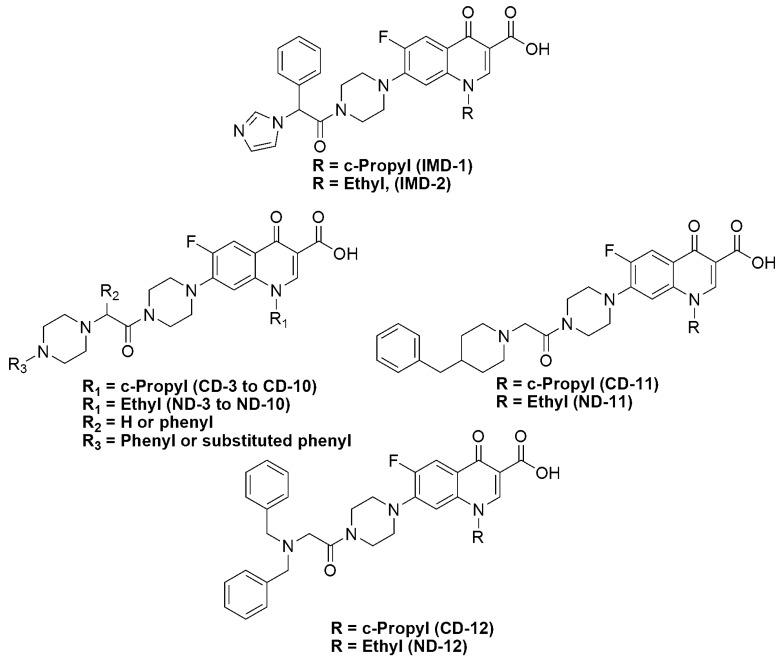
The general structures of the proposed compounds.

Preliminary results in our labs showed that the α-imidazolyl *N_4_*-acetamide derivatives of CIPRO and NOR, **IMD-1** and **IMD-2**, have significant antibacterial activity with enhanced Gram-positive selectivity, Figure 3. Although these two compounds were synthesized to test for potential anticandidal activity, this result prompted the design of a series of α-substituted-*N_4_*-acetamide derivatives of CIPRO and NOR. Herein, we report a group of α-(*N*-arylpiperazinyl), α-(4-benzylpiperidinyl) or α-(*N*,*N*-dibenzylamine)-substituted *N_4_*-acetamides of CIPRO and NOR, Figure 3, as potential lead compounds for potent and selective “anti-Gram-positive” agents. In the design of these compounds, a new basic nitrogen was introduced at the α-carbon of the acetamide, because acetylation rendered the *N_4_* of the piperazine at C7 non-basic. In addition, the introduced aromatic systems were chosen to have relatively diverse electronic and lipophilic characteristics.

The synthesized compound were assayed for antibacterial activity against *Pseudomonas aeruginosa* ATCC 9027, *Escherichia coli* ATCC 8739, *Staphylococcus aureus* ATCC 6538P and *Bacillus subtilis* ATCC 6633. In addition, four quantitative structure activity relationship (QSAR) models that describe the antibacterial activity of these compounds against *S. aureus* and *B. subtilis* were obtained using Partial least squares (PLS) regression. One important feature of these models was the use of ^13^C-NMR data as one of the 2D QSAR descriptors [22,23]. There is always a quest among medicinal chemists to design new antibacterial agents that are effective against resistant strains. Although this is a legitimate and important goal, little is done to develop new antibacterial agents that do not contribute to the rapid emergence of resistant strains. It could be argued that developing narrow spectrum antibacterial agents, like the work presented in this report, will be one step in that direction.

## 2. Results and Discussion

### 2.1. Chemistry

**Scheme 1:**
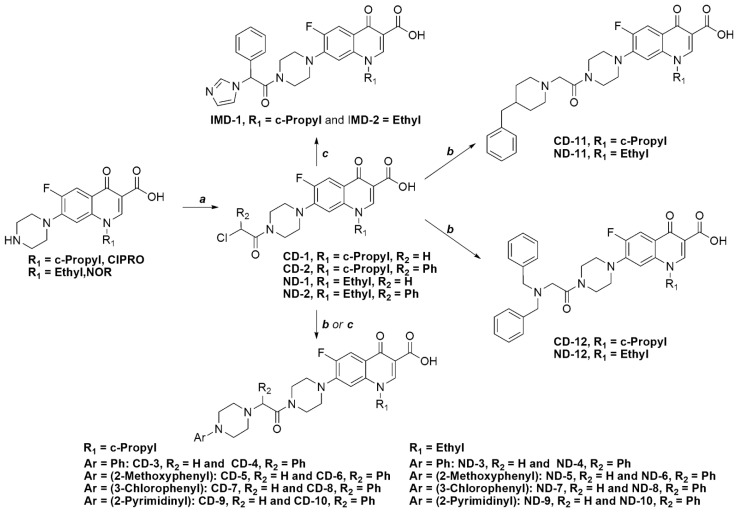
The synthetic scheme for the target compounds. Reaction conditions: (**a**) 2-chloroacetyl chloride or 2-chloro-2-phenylacetyl chloride, Et_3_N, THF, r.t.; (**b**) appropriate amine, Et_3_N, NaI, acetonitrile, r.t.; and (**c**) appropriate amine, Et_3_N, NaI, acetonitrile, reflux.

Scheme 1 illustrates the synthetic pathways of all target compounds. The α-chloroacetamides, **CD-1**, **CD-2**, **ND-1** and **ND-2**, were obtained by treating CIPRO or NOR with 2-chloroacetyl chloride or 2-chloro-2-phenylchloroacetyl chloride in tetrahydrofuran in the presence of triethylamine as a base at room temperature, and the resultant compounds were crystallized from acetonitrile. The α-chloroacetamides, **CD-2** and **ND-2**, were treated with imidazole to afford **IMD-1** and **IMD-2**, respectively. On the other hand, all of the α-chloroacetamides, **CD-1**, **CD-2**, **ND-1** and **ND-2**, were reacted with the appropriate arylpiperazine in the presence of triethylamine and sodium iodide to afford compounds **CD-3** to **CD-10** and **ND-3** to **ND-10**. These reactions were carried out in acetonitrile at room temperature for **CD-1** and **ND-1** and at reflux for **CD-2** and **ND-2**. 4-Benzylpiperidine and dibenzylamine were coupled to **CD-1** and **ND-1** only, as the substitution reaction failed with bulkier **CD-2** and **ND-2**, even when the reaction was maintained at reflux for several days, which might be due to the increased bulk of the nucleophile, at least in the case of dibenzylamine. The reaction with **CD-1** and **ND-1** proceeded in the presence of triethylamine and sodium iodide in acetonitrile at room temperature to afford compounds **CD-11**, **CD-12**, **ND-11** and **ND-12**. From a spectral point of view, the well-known characteristic splitting of the signals corresponding to C5, C6 and C7 of the fluoroquinolone nucleus with C–F coupling constants (*J*) of about 22, 246 and 10 Hz, respectively, was evident in all of the ^13^C-NMR spectra [24].

### 2.2. In Vitro Antibacterial Activity Assays

The final compounds and the intermediates, **CD-2** and **ND-2**, were tested against four standard strains of *P. aeruginosa*, *E. coli*, *S. aureus* and *B. subtilis*. CIPRO and NOR were used as reference compounds. Susceptibility testing assays were performed according to the broth microdilution standard method of the Clinical and Laboratory Standards Institute, CLSI. Table 1 shows the MIC values in μM and μg/mL although the μM, only, will be used for SAR and QSAR interpretation.

In general, the tested compounds were more selective against the Gram-positive *S. aureus* and *B. subtilis*. With regard to the CIPRO derivatives, compound **CD-10** (α-phenyl, Ar = 2-pyrimidyl) exhibited the best activity against *S. aureus* with MIC = 0.4 μM compared to 2.2 μM for CIPRO. The CIPRO derivatives, **CD-7** (Ar = 3-Chlorophenyl) and **CD-9** (Ar = 2-pyrimidyl), were also more active against *S. aureus* than CIPRO and exhibited MIC values of 0.6 and 0.9 μM, respectively. On the other hand, compound **CD-10** exhibited the best activity against *B. subtilis* with MIC = 0.1 μM compared to 0.3 μM for CIPRO. Compounds **CD-5** (Ar = 2-methoxyphenyl), **CD-6** (α-phenyl, Ar = 2-methoxyphenyl), **CD-7** (Ar = 3-chlorophenyl), **CD-8** (α-phenyl, Ar = 3-chlorophenyl) and **CD-9** (Ar = 2-pyrimidyl), although less active than CIPRO, showed MIC values in the range of 0.3–0.9 μM against *B. subtilis.*

Although none of the NOR derivatives showed better activity against *S. aureus* than NOR itself (MIC = 1.1 μM), compounds **ND-6** (α-phenyl, Ar = 2-methoxyphenyl) and **ND-7** (Ar = 3-chlorophenyl) showed MIC values that were less than 2 μM. On the other hand, compounds **ND-6**, **ND-7**, **ND-8** (α-phenyl, Ar = 3-chlorophenyl) and **ND-10** (α-phenyl, Ar = 2-pyrimidyl) exhibited MIC values in the range of 0.6–1.0 μM against *B. subtilis* compared to 0.6 μM for NOR. Finally, it is worth mentioning that all of the compounds possessed lower activity against Gram-negative bacteria compared to the reference drugs.

**Table 1:** The MIC values (μM) for compounds **IMD-1**, **IMD-2**, **CD-2** to **CD-12** and **ND-2** to **ND-12** and the reference compounds, ciprofloxacin (CIPRO) and norfloxacin (NOR).

ID	MIC							
	*E. coli*		*P. aeruginosa*		*S. aureus*		*B. subtilis*	
	(μM)	(µg/mL)	(μM)	(µg/mL)	(μM)	(µg/mL)	(μM)	(µg/mL)
**IMD-1**	8.5	4.4	114	59	0.6	0.3	0.5	0.2
**CD-2**	7.5	3.7	101	49	0.3	0.2	0.1	0.1
**CD-3**	146	78	733	391	55	29	5.3	2.9
**CD-4**	192	117	2,306	1,406	56	34	5.0	3.1
**CD-5**	24	14	NA *	NA *	6.1	3.4	0.5	0.3
**CD-6**	31	20	366	234	3.1	2.0	0.5	0.3
**CD-7**	9.5	5.4	103	59	0.5	0.3	0.3	0.2
**CD-8**	NA *	NA *	NA *	NA *	9.1	5.9	0.5	0.3
**CD-9**	11	6.1	291	156	0.9	0.6	0.9	0.5
**CD-10**	12	7.4	80	49	0.4	0.2	0.1	0.1
**CD-11**	9.0	4.9	62	34	16.4	9.0	2.1	1.2
**CD-12**	206	117	NA *	NA *	120	68	5.4	3.1
**CIPRO**	1.7	0.6	0.5	0.2	2.2	0.7	0.3	0.1
**IMD-2**	542	273	775	NA *	4.7	2.4	1.2	0.6
**ND-2**	10	4.9	103	NA *	0.4	0.2	0.1	0.1
**ND-3**	112	58	1,198	NA*	23	12	3.5	1.8
**ND-4**	NA *	NA *	NA *	NA *	392	234	9.2	5.5
**ND-5**	1,133	625	NA *	NA *	42	23	8.8	4.9
**ND-6**	NA *	NA *	NA *	NA *	1.9	1.2	0.7	0.4
**ND-7**	NA *	NA *	NA *	NA *	1.8	1.0	0.6	0.3
**ND-8**	NA *	NA *	NA *	NA *	4.6	2.9	1.0	0.6
**ND-9**	130	68	3,581	1,875	13	7.0	3.7	2.0
**ND-10**	81	49	1,042	625	2.5	1.5	0.8	0.5
**ND-11**	165	88	NA *	NA *	7.1	3.8	4.6	2.4
**ND-12**	105	59	983	547	123	68	5.5	3.1
**NOR**	2.3	0.7	0.6	0.2	1.1	0.4	0.6	0.2

* No antibacterial activity was found at the highest concentration used in this assay.

### 2.3. Structure-Activity Relationships

Due to the low antibacterial activity of these compounds against Gram-negative bacteria, which is one of the goals of this work, the current discussion of structure-activity relationships (SAR) will be confined to their activity against Gram-positive bacteria.

The extraction of useful SAR from the above results was a complicated task. Nevertheless, the effect of the α-phenyl group was an interesting aspect in the SAR of these compounds. For the CIPRO derivatives, the presence of the α-phenyl did not affect the activity of **CD-3** compared to **CD-4** (Ar = phenyl) and **CD-5** compared to **CD-6** (Ar = 2-methoxyphenyl). In contrast, the presence of the α-phenyl group had a marked effect on the activity of the least polar (Ar = 2-chlorophenyl) and the most polar (Ar = 2-pyrimidyl) derivatives, but in opposite directions. It can be seen that compound **CD-7** showed higher activity compared to **CD-8** (Ar = 2-chlorophenyl), and compound **CD-9** showed lower activity compared to **CD-10** (Ar = 2-pyrimidyl), *i.e.*, the less polar derivative becomes less active and the more polar derivatives becames more active.

With regard to the NOR derivatives, the activity of all the compounds were affected by the α-phenyl group. Compound **ND-3** showed higher activity than **ND-4** (Ar = phenyl). **ND-5** showed lower activity than **ND-6** (Ar = 2-methoxyphenyl). **ND-7** showed much higher activity than **ND-8** (Ar = 2-chlorophenyl), and **ND-9** showed much lower activity than **ND-10** (Ar = 2-pyrimidyl). Again, the presence of the α-phenyl caused the less polar derivative (Ar = phenyl or Ar = = 2-chlorophenyl) to become less active and the more polar derivatives (Ar = 2-methoxyphenyl or Ar = 2-pyrimidyl) to became more active. Regardless of the differences in activity between in the CIPRO and NOR groups, it seems that a critical balance between polarity and lipophilicity is at play in the SAR of this group of compounds.

In addition to increasing the lipophilic character, the presence of the α-phenyl group also introduced a stereogenic center, which meant that all of the α-phenyl-containing compounds are racemic mixtures. Although it is well known that the activity of pure enantiomers can be different in magnitude and, sometimes, kind, the authors believe that tedious enantiomeric resolution or elaborate stereoselective syntheses at this stage of lead identification is not warranted.

With regard to **CD-12** and **ND-12**, the presence of a dibenzylamine moiety led to derivatives with generally low activity, which might be due to their increased flexibility and/or bulk.

Finally, it is worth mentioning that **CD-5** was shown to exhibit antiproliferative activity against breast cancer cells and melanoma by inducing apoptosis by the generation of reactive oxygen species [25].

### 2.4. Quantitative Structure-Activity Relationships (QSAR)

Despite the previously discussed balance between polarity and lipophilicity, the MIC values of the synthesized compounds did not correlate quantitatively with their calculated Log distribution coefficient (cLog D) values, and no useful quantitative models were obtained by plotting the activity against clog D. This lack of correlation has been reported previously in the literature [26,27]. Hence, a more elaborate QSAR model was sought in which the activity was correlated with a small set of independent variables composed of clog D, molecular fractional polar surface area (FPSA) and Δ_C-13_ (DMSO-*d_6_*). Table 2 shows the values of these variables, in addition to the values of the dependent variable, log 1/MIC (MIC in µM).

cLog D was chosen instead of log P (log partition coefficient), because the former takes into consideration the ionizable nature of the compounds under investigation at pH 7.4 [28]. ^13^C-NMR was included in the computation of these models, because it has been shown that constructing QSAR models “using chemical shifts of ^13^C-NMR works very well when attempted on a set of compounds with a large proportion of carbon nuclei or on similar structural motifs” [22]. There are undeniable advantages to using the ^13^C-NMR in QSAR studies. First and foremost, the spectra are acquired in solution, which is a close simulation to biological systems. The second is that the chemical shifts in ^13^C-NMR are very sensitive to the molecular connectivity and shape [22]. In addition, ^13^C-NMR chemical shifts are the result of experiment, whereas most of the independent descriptors used to construct QSAR models are estimated values. Only recently, ^13^C-NMR chemical shifts were used successfully to study the property-property and property-drug likeness relationships of some fluoroquinolone salts [23]. Furthermore, the authors are not aware of any QSAR reports on fluoroquinolones that involved the use of ^13^C-NMR chemical shifts as 2D descriptors. The molecular fractional polar surface area was chosen because it reflects the molecule’s polarity, which is an important determinant of its ability to penetrate biological membranes [29], which is very important, especially since it is believed that fluoroquinolones accumulates in *S. aureus* by simple diffusion [30].

**Table 2:** Log (1/MIC), clog D, molecular fractional polar surface area (FPSA) and **∆_C-13_** for compounds **IMD-1**, **IMD-2**, **CD-2** to **CD-12** and **ND-2** to **ND-12**.

ID	Log (1/MIC)		cLog D	FPSA	∆_C-13_ *
	*S. aureus*	*B. subtilis*			
**IMD-1**	0.229	0.328	2.216	0.216	0.550
**CD-2**	0.523	1.000	2.009	0.189	−0.480
**CD-3**	−1.739	−0.728	1.359	0.185	1.690
**CD-4**	−1.746	−0.699	3.111	0.163	2.770
**CD-5**	−0.783	0.268	1.371	0.191	1.770
**CD-6**	−0.486	0.319	3.094	0.170	2.250
**CD-7**	0.268	0.495	2.052	0.177	1.650
**CD-8**	−0.968	0.319	3.775	0.156	2.740
**CD-9**	0.041	0.041	−1.433	0.236	1.630
**CD-10**	0.444	1.000	0.286	0.208	−0.510
**CD-11**	−1.215	−0.330	0.859	0.173	2.030
**CD-12**	−2.079	−0.729	1.469	0.166	2.510
**IMD-2**	−0.675	−0.086	1.840	0.210	0.40
**ND-2**	0.387	1.000	3.337	0.187	−0.660
**ND-3**	−1.362	−0.545	1.670	0.177	1.500
**ND-4**	−2.593	−0.962	4.401	0.168	2.590
**ND-5**	−1.620	−0.946	1.877	0.183	1.570
**ND-6**	−0.288	0.168	4.385	0.168	2.650
**ND-7**	−0.246	0.260	2.558	0.176	1.470
**ND-8**	−0.667	0.013	5.066	0.155	2.570
**ND-9**	−1.127	−0.571	−1.245	0.234	1.490
**ND-10**	−0.398	0.092	0.496	0.206	2.630
**ND-11**	−0.848	−0.659	1.045	0.172	1.690
**ND-12**	−2.089	−0.739	3.276	0.165	2.240

* (DMSO-*d_6_*).

**Figure 4:**
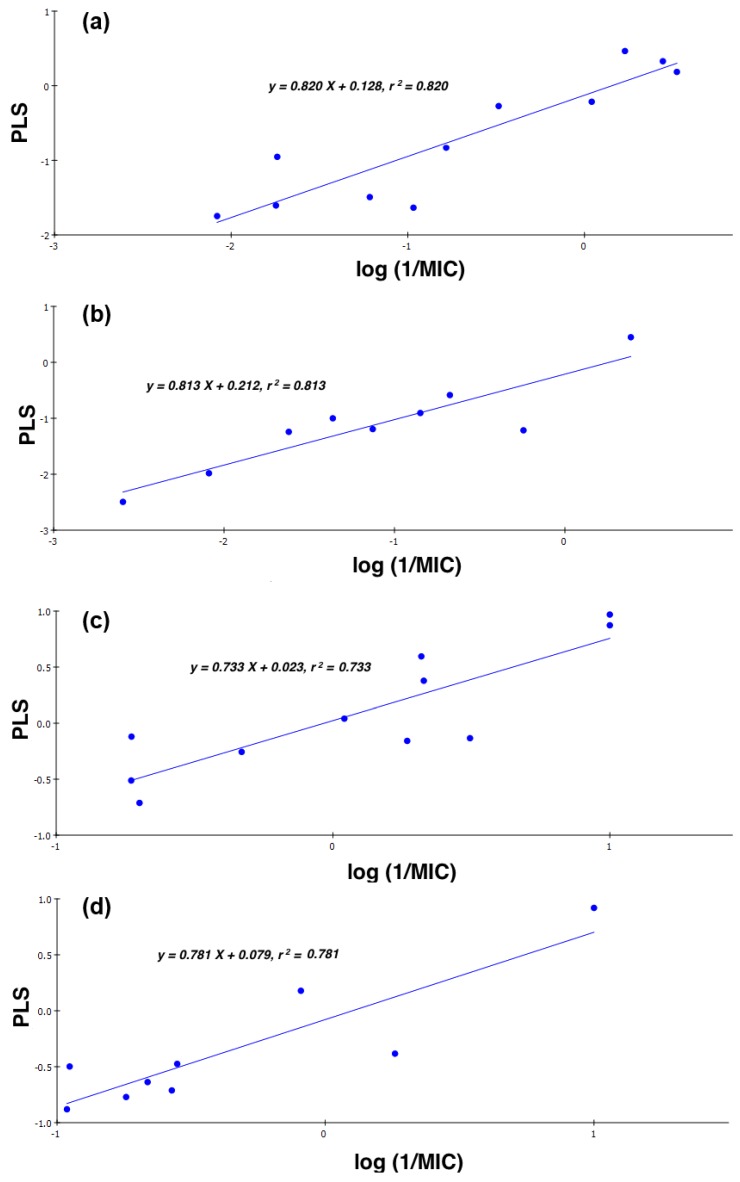
The Partial least squares (PLS) regression models for (**a**) CD-S: CIPRO derivatives against *S. aureus*; (**b**) ND-S: NOR derivatives against *S. aureus*; (**c**) CD-B: CIPRO derivatives against *B. subtilis*; and (**d**) ND-B: NOR derivatives against *B. subtilis*.

Four models were obtained, Figure 4, CD-S and ND-S, which described the activity of the CIPRO and NOR derivatives against *S. aureus*, respectively and CD-B and ND-B, which described the activity of the CIPRO and NOR derivatives against *B. subtilis*, respectively. These models were as follows:

(1) CD-S: Log (1/MIC) = −5.107 + 0.198 cLogD + 24.830 FPSA − 0.420 ∆_C-13_, *n* = 11, *r* = 0.905, *r^2^* = 0.820, RMSEE = 0.377, *q^2^* = 0.636 and MAE = 0.474
(2) ND-S: Log (1/MIC) = 4.315 − 0.298 cLogD − 18.840 FPSA − 0.988 ∆_C-13_, *n* = 9, *r* = 0.902, *r^2^* = 0.813, RMSEE = 0.374, *q^2^* = 0.594 and MAE = 0.939
(3) CD-B: Log (1/MIC) = 0.741 − 4.735 × 10^−2^ cLogD + 5.169 × 10^−3^ FPSA − 0.472 ∆_C-13_, *n* = 11, *r* = 0.856, *r^2^* = 0.733, RMSEE = 0.316, *q^2^* = 0.681 and MAE = 0.260
(4) ND-B: Log (1/MIC) = 0.257 + 8.340 × 10^−2^ cLogD + 4.373 × 10^−3^ FPSA − 0.581 ∆_C-13_, *n* = 9, *r* = 0.884, *r^2^* = 0.781, RMSEE = 0.285, *q^2^* = 0.699 and MAE = 0.471

where *r* is the regression coefficient, *r^2^* is the non-cross-validated variance of the coefficient, *q*^2^ is the cross-validated variance of the coefficient, RMSEE is the root mean square error of regression and MAE is the mean absolute error of cross-validation. All of the models had acceptable linearity (*r^2^*, 0.733–0.820) and validity (*q^2^*, 0.594–0.699).

From the QSAR equations, it is clear that activity against *S. aureus* is highly affected by FPSA. For the CD-S model, the sign of the coefficient for FPSA is positive, indicating that activity is proportional to polarity, while the sign is negative in the model corresponding to ND-S, indicating that the activity is decreased by increasing polarity. Actually, the previous interpretation alone would be misleading if another two important factors were not taken into consideration. First, the values for FPSA are at least one order of magnitude less than the cLog D values. Second, the effect of lipophilicity (clog D) balances that of polarity (FPSA) in these two models. In addition, the effect of electron density in the vicinity of the acetamide moiety cannot be neglected, as the log (1/MIC) is negatively affected by Δ_C-13_. Here, Δ_C-13_ is the difference in ^13^C-NMR chemical shifts (δ) between the peak corresponding to the carbonyl carbons of the amide and the carboxylic acid groups, δ_amide_ − δ_acid_ (Δ_C-13_). A positive Δ_C-13_, in general, indicates that the environment around the α-acetamide carbonyl carbon is electron deficient, while a negative Δ_C-13_ indicates the opposite. Since the coefficient of Δ_C-13_ is negative, this means that compounds with higher electron density will exhibit higher activity. The resultant effect of these three factors is what makes these models logical. This variation in the dependency of the antibacterial activity of the CIPRO derivatives *vs.* the NOR derivatives on polarity is not totally surprising, because it has been previously established that the intracellular activity of fluoroquinolones is influenced differently for each of the different molecules [31].

With regard to antibacterial activity against *B. subtilis*, it seems to be highly dependent on Δ_C-13_ with a negative sign for the coefficient, indicating that higher electron density around the acetamide substituents will result in higher activity against *B. subtilis*. While the effect of FPSA in these models was minimal, cLog D had a significant effect. For the CIPRO derivatives, lower lipophilicity is desired, and for the NOR derivatives, higher lipophilicity is better. Actually, the same trend is applicable for CD-S and ND-S, although as seen before, this effect was less than that for FPSA. It is interesting to see, again, that the activity of different fluoroquinolone nuclei is affected differently by certain variables. Finally, it can be noticed that there were more outliers in the models obtained for the NOR derivatives than the CIPRO derivatives, regardless of the microorganism in question. One possible explanation is the fact that cLog D values for the NOR derivatives are higher than those of the CIPRO derivatives, while other descriptors remained very similar.

These QSAR models are independent of stereochemistry, because all of the used descriptors are expected to be identical for enantiomers.

## 3. Experimental

### 3.1. Chemistry

Bulk solvents were purchased through local vendors. Reagent-grade and fine chemicals were obtained from, Aldrich Chemical Company (St. Louis, MO, USA), ACROS Chemicals (Geel, Belgium) and Scharlau Chemical (Barcelona , Spain). Melting points were determined using a Stuart Scientific melting point apparatus (Stuart Scientific, Stone, Staffordshire, UK) and were uncorrected. IR spectra were recorded on an IRAffinity-1 FT-IR (Shimadzu, Kyoto, Japan) using KBr disks, and the absorptions are reported in cm^−1^. NMR spectra were obtained with a Bruker Advance Ultrashield 400 MHz instrument (Bruker, Fallanden, Switzerland), and chemical shifts (δ) are reported in ppm relative to automatic calibration to the residual proton peak of the solvent used, namely CDCl_3_ or DMSO-*d*_6_. TLC analysis was performed on Merck aluminum TLC plates, Silica 60, F_254_ (Merck, Darmstadt, Germany). Mass spectra were obtained by an Agilent 1100 series LC-MSD-Trap instrument using Atmospheric Pressure Chemical Ionization, ACPI (Agilent, Santa Clara, USA). HRMS data were obtained on a Bruker APEX-4, 7 Tesla (Bruker, Bremen, Germany).

### 3.2. General Procedures for the Synthesis of Intermediate Compounds(***CD-1***, ***CD-2***, ***ND-1*** and ***ND-2***)

To a 250-mL round-bottomed flask containing a mixture of CIPRO or NOR (1 equivalents) and tetrahydrofuran (100 mL), triethylamine (1.3 equivalents) was added, followed by the slow addition of chloroacetyl chloride or 2-chloro-2-phenylacetyl chloride (1.3 equivalents). The reaction mixture was stirred for 10–15 min at room temperature, and it was followed up by TLC (10% methanol/dichloromethane). The solvent was then evaporated, and the residue was suspended in saturated NaCl solution; then, the compound was extracted with dichloromethane. The organic layer was dried over Na_2_SO_4_, then evaporated, and the compound was crystallized from acetonitrile.

#### 3.2.1. 7-(4-(2-Chloroacetyl)piperazin-1-yl)-1-cyclopropyl-6-fluoro-4-oxo-1,4-dihydro-quinoline-3-carboxylic acid (**CD-1**)

Ciprofloxacin (3 g, 9 mmol); chloroacetyl chloride (0.93 mL, 11.77 mmol) and triethylamine (1.64 mL, 11.77 mmol). The yield of **CD-1** was 54% of a pale-yellow powder; m.p. 266–269 °C; IR, cm^−1^, (KBr): 3072–3010 (Ar-H), 2922–2854 (–CH), 1728 (C=O, acid), 1660 (C=O, amide), 1627 (C=O, ketone), 1541–1438 (C=C); LC-MS (APCI) *m/z*: 408 ([M+H]^+^, 100%); HRMS *m/z* calculated: 408.11264, found: 408.11209; ^1^H-NMR (400 MHz, DMSO-*d*_6_): δ 1.15–1.34 (m, 4H, cyclopropyl); 3.38 (bs, 4H, piperazine); 3.68 (bs, 4H, piperazine); 3.80 (tt, 1H, cyclopropyl); 4.45 (s, 2H, COCH_2_Cl); 7.54 (d, 1H, H-8 of quinolone, *J*_H-F_ = 7 Hz); 7.85 (d, 1H, H-5 of quinolone, *J*_H-F_ = 13 Hz); and 8.62 (s, 1H, H-2 of quinolone). ^13^C-NMR (100 MHz, DMSO-*d*_6_): δ 12.1, 40.3, 45.8, 46.3, 49.5, 53.5, 53.8, 111.1, 111.2, 115.5 (*J*_C-F_ = 22 Hz), 123.3, 143.5, 149.3 (*J*_C-F_ = 10 Hz), 152.5, 157.1 (*J*_C-F_ = 248 Hz), 169.3, 170.3 and 180.8.

#### 3.2.2. 7-(4-(2-Chloroacetyl)piperazin-1-yl)-1-ethyl-6-fluoro-4-oxo-1,4-dihydroquinoline-3-carboxylic acid (**ND-1**)

Norfloxacin (3 g, 9.4 mmol); chloroacetyl chloride (0.97 mL, 12.21 mmol) and triethylamine (1.70 mL, 12.21 mmol). The yield of **ND-1** was 32% of a light-brown powder; m.p. 261–263 °C; IR, cm^−1^, (KBr): 3049–2964 (Ar-H), 2910–2858 (–CH), 1720 (C=O, acid), 1653 (C=O, amide), 1625 (C=O, ketone), 1552–1436 (C=C); LC-MS (APCI) *m/z*: 396 ([M+H]^+^, 100%); HRMS *m/z* calculated: 396.11264, found: 418.09403 (M+Na^+^); ^1^H-NMR(400 MHz, DMSO-*d*_6_): δ 1.40 (t, 3H, CH_3_, *J*_H-H_ = 7 Hz); 3.36 (bs, 4H, piperazine); 3.67 (bs, 4H, piperazine); 4.44 (s, 2H, COCH_2_Cl); 4.57 (q, 2H, CH_2_Me, *J*_H-H_ = 7 Hz); 7.17 (d, 1H, H-8 of quinolone, *J*_H-F_ = 7 Hz); 7.90 (d, 1H, H-5 of quinolone, *J*_H-F_ = 13 Hz); and 8.92 (s, 1H, H-2 of quinolone). ^13^C-NMR (100 MHz, DMSO-*d*_6_): δ 18.9, 45.8, 46.3, 49.5, 53.6, 53.7, 53.9, 110.7, 111.6, 115.7 (*J*_C-F_ = 23 Hz), 124.1, 141.6, 149.6 (*J*_C-F_ = 10 Hz), 153.4, 157.3 (*J*_C-F_ = 247 Hz), 169.3, 170.6 and 180.6.

#### 3.2.3. 7-(4-(2-Chloro-2-phenylacetyl)piperazin-1-yl)-1-cyclopropyl-6-fluoro-4-oxo-1,4-dihydroquinoline-3-carboxylic acid (**CD-2**)

Ciprofloxacin (2 g, 6 mmol); 2-chloro-2-phenylacetyl chloride (1.47 g, 7.8 mmol) and triethylamine (1.09 mL, 7.82 mmol). The yield of **CD-2** was 45% of pale-yellow powder; m.p. 156–159 °C; IR, cm^−1^, (KBr): 3064–3008 (Ar-H), 2918–2837 (–CH), 1724 (C=O, acid), 1658 (C=O, amide), 1629 (C=O, keto), 1544–1388 (C=C); LC-MS (APCI) *m/z*: 484 ([M+H]^+^, 100%); HRMS *m/z* calculated: 484.14339, found: 484.4394; ^1^H-NMR(400 MHz, DMSO-*d*_6_): δ 1.12–1.29 (m, 4H, cyclopropyl); 3.04–3.30 (m, 4H, piperazine); 3.60–3.78(m, 1H, cyclopropyl and 4H, piperazine); 6.42 (s, 1H, COCHClPh); 7.35 (d, 1H, H-8 of quinolone); 7.35–7.53 (m, 5H, Ar-H); 7.86 (d, 1H, H-5 of quinolone, *J*_H-F_ = 13 Hz); and 8.63 (s, 1H, H-2 of quinolone). ^13^C-NMR (100 MHz, DMSO-*d*_6_): δ 8.1, 36.4, 42.4, 45.6, 49.5, 49.8, 57.9, 107.2, 107.3, 111.5 (*J*_C-F_ = 23 Hz), 119.4, 128.8, 129.3, 129.4, 137.5, 139.6, 145.2 (*J*_C-F_ = 10 Hz), 148.6, 153.3 (*J*_C-F_ = 248 Hz), 165.9, 166.4 and 176.9.

#### 3.2.4. 7-(4-(2-Chloro-2-phenylacetyl)piperazin-1-yl)-1-ethyl-6-fluoro-4-oxo-1,4-dihydroquinoline-3-carboxylic acid (**ND-2**)

Norfloxacin (2 g, 6.3 mmol); 2-chloro-2-phenylacetyl chloride (1.54 g, 8.14 mmol) and triethylamine (1.14 mL, 8.18 mmol). The yield of **ND-2** was 44% of yellow powder; m.p. 177–180 °C; IR, cm^−1^, (KBr): 3050–2950 (Ar-H), 2924–2840 (–CH), 1724 (C=O, acid), 1658 (C=O, amide), 1627 (C=O, keto), 1506–1440 (C=C); LC-MS (APCI) *m/z*: 472 ([M+H]^+^, 100%); HRMS *m/z* calculated: 472.14394, found: 472.14339; ^1^H-NMR(400 MHz, DMSO-*d*_6_): δ 1.38 (t, 3H, CH_3_, *J*_H-H_ = 7 Hz); 3.06–3.34 (m, 4H, piperazine); 3.63–3.81 (m, 4H, piperazine); 4.55 (q, 2H, CH_2_Me, *J*_H-H_ = 7 Hz); 6.42 (s, 1H, COCHClPh); 7.12 (d, 1H, H-8 of quinolone, *J*_H-F_ = 7 Hz); 7.36–7.53 (m, 5H, Ar-H); 7.88 (d, 1H, H-5 of quinolone, *J*_H-F_ = 13 Hz); and 8.92 (s, 1H, H-2 of quinolone). ^13^C-NMR (100 MHz, DMSO-*d*_6_): δ 15.4, 41.5, 42.9, 46.1, 50.1, 50.3, 58.3, 107.1, 108.1, 112.2 (*J*_C-F_ = 28 Hz), 120.5, 129.3, 129.7, 129.7, 137.9, 138.1, 146.0, 149.5 (*J*_C-F_ = 11 Hz), 153.8 (*J*_C-F_ = 246 Hz), 166.4, 167.1 and 177.1.

### 3.3. General Procedure for the Synthesis of Final Compounds

Method A: To a solution of intermediates **CD-1** or **ND-1** (1 equivalent) in acetonitrile (40 mL), triethylamine (2–6 equivalent), the proper secondary amine (2–3 equivalents) and sodium iodide (1 equivalent) were added. The reaction mixture was allowed to stir at room temperature for 16–20 h. The reaction was followed up with TLC with a suitable mobile phase. The solvent was evaporated, and the residue was dissolved in dichloromethane, then washed with saturated NaCl solution (50 mL, 3 times). The organic layer was dried over Na_2_SO_4_, then evaporated, and the residue was crystallized from suitable solvent.

Method B: The same as Method A, but intermediates **CD-2** or **ND-2** were used and the reaction mixtures maintained under reflux for 20–24 h.

#### 3.3.1. 1-Cyclopropyl-6-fluoro-7-(4-(2-(1H-imidazol-1-yl)-2-phenylacetyl)piperazin-1-yl)-4-oxo-1,4-dihydroquinoline-3-carboxylic acid (**IMD-1**)

Method B: Compound **CD-2** (0.5 g, 1.0 mmol); imidazole (0.20 g, 3 mmol) and triethylamine (0.42 mL, 3 mmol). **IMD-1** was recrystallized from ethanol to give 18% yield of pale-yellow crystals; m.p. 171–174 °C; IR, cm^−1^, (KBr): 3150–3000 (Ar-H), 2920–2800 (–CH), 1715 (C=O, acid), 1650 (C=O, amide), 1627 (C=O, keto), 1508–1456 (C=C); LC-MS (APCI) *m/z*: 516 ([M+H]^+^, 100%); ^1^H-NMR(400 MHz, DMSO-*d*_6_): δ 1.12–1.26 (m, 4H, cyclopropyl); 2.90 (m, 1H, piperazine); 3.23–3.37 (m, 1H, cyclopropyl and 3H, piperazine); 3.74–3.77 (m, 4H, piperazine); 6.85 (s, 1H, imidazole); 6.87 (s, 1H, imidazole); 7.12 (s, 1H, COCHPh); 7.38–7.46 (m, 5H, Ar-H); 7.56 (d, 1H, H-8 of quinolone, *J*_H-F_ = 9 Hz); 7.64 (s, 1H, imidazole) 7.77–7.81 (d, 1H, H-5 of quinolone, *J*_H-F_ = 13 Hz); and 8.59 (s, 1H, H-2 of quinolone). ^13^C-NMR (100 MHz, DMSO-*d_6_*): δ 8.5, 36.8, 42.5, 45.8, 49.9, 60.7, 107.4, 107.6, 111.9 (*J*_C-F_ = 24 Hz), 119.7, 120.2, 128.6, 129.1, 129.8, 130.3, 136.6, 138.1, 139.9, 145.5 (*J*_C-F_ = 10 Hz), 148.9, 153.7 (*J*_C-F_ = 248 Hz), 166.8, 167.3 and 177.2.

#### 3.3.2. 1-Ethyl-6-fluoro-7-(4-(2-(1H-imidazol-1-yl)-2-phenylacetyl)piperazin-1-yl)-4-oxo-1,4-dihydroquinoline-3-carboxylic acid (**IMD-2**)

Method B: Compound **ND-2** (0.5 g, 1.1 mmol); imidazole (0.22 g, 3.3 mmol) and triethylamine (0.46 mL, 3.3 mmol). **IMD-2** was crystallized from ethanol to give 16% yield of pale-yellow crystals; m.p. 163–166 °C; IR, cm^−1^, (KBr): 3120–3000 (Ar-H), 2980–2850 (–CH), 1716 (C=O, acid), 1653 (C=O, amide), 1627 (C=O, keto), 1500–1450 (C=C); LC-MS (APCI) *m/z*: 504 ([M+H]^+^, 100%); ^1^H-NMR (400 MHz, DMSO-*d*_6_): δ 1.34 (t, 3H, CH_3_, *J*_H-H_ = 7 Hz); 2.95 (m, 1H, piperazine); 3.25–3.32 (m, 3H, piperazine); 3.72–3.75 (m, 4H, piperazine); 4.53 (q, 2H, CH_2_Me, *J*_H-H_ = 7 Hz); 6.84 (s, 1H, imidazole); 6.86 (s, 1H, imidazole); 7.08 (d, 1H, H-8 of quinolone, *J*_H-F_ = 7 Hz); 7.12 (s, 1H, COCHPh); 7.38–7.57 (m, 5H, Ar-H); 7.64 (s, 1H, imidazole) 7.84 (d, 1H, H-5 of quinolone, *J*_H-F_ = 13 Hz); and 8.91 (s, 1H, H-2 of quinolone). ^13^C-NMR (100 MHz, DMSO-*d*_6_): δ 15.4, 41.1, 41.1, 41.2, 42.6, 45.9, 50.1, 60.7, 107.1, 108.0, 112.3 (*J*_C-F_ = 23 Hz), 120.5, 120.8, 128.7, 129.1, 129.9, 130.3, 136.8, 138.1, 138.2, 145.9 (*J*_C-F_ = 10 Hz), 149.6, 153.7 (*J*_C-F_ = 248 Hz), 167.0, 167.4 and 177.1.

#### 3.3.3. 1-Cyclopropyl-6-fluoro-4-oxo-7-(4-(2-(4-phenylpiperazin-1-yl)acetyl)piperazin-1-yl)-1,4-dihydroquinoline-3-carboxylic acid (**CD-3**)

Method A: Compound **CD-1** (0.5 g, 1.2 mmol); phenylpiperazine (0.37 mL, 2.4 mmol) and triethylamine (0.33 mL, 2.4 mmol). **CD-3** was crystallized from acetonitrile to give 21% yield of off-white crystals; m.p. 240–242 °C; IR, cm^−1^, (KBr): 3100–3000 (Ar-H), 2933–2818 (–CH), 1718 (C=O, acid), 1654 (C=O, amide), 1629 (C=O, keto), 1541–1440 (C=C); LC-MS (APCI) *m/z*: 534 ([M+H]^+^, 100%); HRMS *m/z* calculated: 534.25166, found: 534.25111; ^1^H-NMR(400 MHz, DMSO-*d_6_*): δ 1.08–1.27 (m, 4H, cyclopropyl); 2.49 (bs, 2H, piperazine); 2.58 (bs, 2H, piperazine); 3.13 (bs, 4H, piperazine) 3.27 (s, 2H, COCH_2_–); 3.33 (bs, 4H, piperazine); 3.69 (bs, 2H, piperazine); 3.80 (bs, 2H, piperazine); 3.80 (m, 1H, cyclopropyl); 6.75 (t, 1H, p-Ar-H, *J*_H-H_ = 7 Hz); 6.91 (d, 2H, o-Ar-H, *J*_H-H_ = 8 Hz); 7.19 (t, 2H, m-Ar-H, *J*_H-H_ = 8 Hz); 7.56 (d, 1H, H-8 of quinolone, *J*_H-F_ = 7 Hz); 7.90 (d, 1H, H-5 of quinolone, *J*_H-F_ = 13 Hz); and 8.64 (s, 1H, H-2 of quinolone). ^13^C-NMR (100 MHz, DMSO-*d_6_*): δ 8.5, 36.2, 41.9, 45.9, 49.2, 50.3, 50.9, 53.4, 61.5, 107.6, 107.7, 112.0 (*J*_C-F_ = 22 Hz), 116.4, 119.7, 119.8, 129.7, 140.2, 146.0 (*J*_C-F_ = 10 Hz), 149.0, 151.9, 154.1 (*J*_C-F_ = 258 Hz), 166.9, 168.6 and 177.5.

#### 3.3.4. 1-Cyclopropyl-6-fluoro-4-oxo-7-(4-(2-phenyl-2-(4-phenylpiperazin-1-yl)acetyl)piperazin-1-yl)-1,4-dihydroquinoline-3-carboxylic acid (**CD-4**)

Method B: Compound **CD-2** (0.5 g, 1.0 mmol); phenylpiperazine (0.31 mL, 2 mmol) and triethylamine (0.28 mL, 2 mmol). **CD-4** was crystallized from acetonitrile to give 40% yield of off-white crystals; m.p. 217–219 °C; IR, cm^−1^, (KBr): 3100–3000 (Ar-H), 2953–2823 (–CH), 1726 (C=O, acid), 1650 (C=O, amide), 1627 (C=O, keto), 1505–1388 (C=C); LC-MS (APCI) *m/z*: 610 ([M+H]^+^, 100%); HRMS *m/z* calculated: 610.28296, found: 610.28241; ^1^H-NMR(400 MHz, CDCl_3_): δ 1.14–1.28 (m, 4H, cyclopropyl); 2.58–2.62 (m, 4H, piperazine); 3.10 (m, 4H, piperazine) 3.24 (bs, 4H, piperazine); 3.64–3.95 (m, 4H, piperazine); 3.75 (m, 1H, cyclopropyl); 4.67 (s, 1H, COCHPh); 6.74 (t, 1H, p-Ar-H, *J*_H-H_ = 7 Hz); 6.87 (d, 2H, o-Ar-H, *J*_H-H_ = 8 Hz); 7.17 (t, 2H, m-Ar-H, *J*_H-H_ = 8 Hz); 7.29–7.48 (m, 5H, Ar-H), 7.30 (d, 1H, H-8 of quinolone, *J*_H-F_ = 7 Hz); 7.87 (d, 1H, H-5 of quinolone, *J*_H-F_ = 13 Hz); and 8.63 (s, 1H, H-2 of quinolone). ^13^C-NMR (100 MHz, CDCl_3_): δ 8.0, 36.3, 41.6, 45.2, 48.8, 49.6, 49.9, 50.5, 69.2, 106.9, 107.2, 111.4 (*J*_C-F_ = 23 Hz), 115.7, 119.1, 119.5, 128.2, 128.8, 129.3, 129.6, 136.3, 139.5, 145.1 (*J*_C-F_ = 12 Hz), 148.4, 151.4, 153.3 (*J*_C-F_ = 245 Hz), 166.3, 169.1 and 176.7.

#### 3.3.5. 1-Ethyl-6-fluoro-4-oxo-7-(4-(2-(4-phenylpiperazin-1-yl)acetyl)piperazin-1-yl)-1,4-dihydroquinoline-3-carboxylic acid (**ND-3**)

Method A: Compound **ND-1** (0.5 g, 1.3 mmol); phenylpiperazine (0.40 mL, 2.6 mmol) and triethylamine (0.36 mL, 2.6 mmol). **ND-3** was crystallized from ethyl acetate/ether to give 24% yield of off- white crystals; m.p. 190–192 °C; IR, cm^−1^, (KBr): 3100–3010 (Ar-H), 2974–2827 (–CH), 1732 (C=O, acid), 1640 (C=O, amide), 1627 (C=O, keto), 1520–1448 (C=C); LC-MS (APCI) *m/z*: 522 ([M+H]^+^, 100%); HRMS *m/z* calculated: 522.25166, found: 522.25111; ^1^H-NMR(400 MHz, DMSO-*d*_6_): δ 1.38 (t, 3H, CH_3_, *J*_H-H_ = 7 Hz); 2.58 (bs, 4H, piperazine); 3.12 (bs, 4H, piperazine); 3.27 (s, 2H, COCH_2_–); 3.30 (bs, 4H, piperazine); 3.64 (bs, 2H, piperazine); 3.79 (bs, 2H, piperazine); 4.56 (q, 2H, CH_2_, *J*_H-H_ = 7 Hz); 6.75 (t, 1H, p-Ar-H, *J*_H-H_ = 7 Hz); 6.90 (d, 2H, o-Ar-H, *J*_H-H_ = 8 Hz); 7.16 (d, 1H, H-8 of quinolone, *J*_H-F_ = 7 Hz); 7.18 (t, 2H, m-Ar-H, *J*_H-H_ = 8 Hz); 7.89 (d, 1H, H-5 of quinolone, *J*_H-F_ = 13 Hz); and 8.92 (s, 1H, H-2 of quinolone). ^13^C-NMR (100 MHz, DMSO-*d*_6_): δ 15.3, 41.9, 45.9, 49.2, 50.0, 50.4, 50.9, 53.4, 61.5, 107.0, 108.0, 112.1 (*J*_C-F_ = 23), 116.3, 119.8, 120.3, 129.8, 138.1, 146.2 (*J*_C-F_ = 10), 149.5, 151.9, 153.8 (*J*_C-F_ = 246 Hz), 167.0, 168.5 and 177.1.

#### 3.3.6. 1-Ethyl-6-fluoro-4-oxo-7-(4-(2-phenyl-2-(4-phenylpiperazin-1-yl)acetyl)piperazin-1-yl)-1,4-dihydroquinoline-3-carboxylic acid (**ND-4**)

Method B: Compound **ND-2** (0.5 g, 1.1 mmol); phenylpiperazine (0.34 mL, 2.2 mmol) and triethylamine (0.31 mL, 2.2 mmol). **ND-4** was crystallized from acetonitrile to give 63% yield of off- white crystals; m.p. 242–244 °C; IR, cm^−1^, (KBr): 3090–3020 (Ar-H), 2960–2829 (–CH), 1714 (C=O, acid), 1650 (C=O, amide), 1624 (C=O, keto), 1525–1429 (C=C); LC-MS (APCI) *m/z:* 598 ([M+H]^+^, 100%); HRMS *m/z* calculated: 598.28296, found: 598.28241; ^1^H-NMR(400 MHz, DMSO-*d*_6_): δ 1.38 (t, 3H, CH_3_, *J*_H-H_ = 7 Hz); 2.58–2.60 (m, 4H, piperazine); 2.80–3.02 (m, 4H, piperazine); 3.10 (bs, 4H, piperazine); 3.26 (bs, 4H, piperazine); 3.61–3.93 (m, 4H, piperazine); 4.55 (q, 2H, CH_2_, *J*_H-H_ = 7 Hz); 4.67 (s, 1H, COCHPh); 6.72–6.76 (t, 1H, p-Ar-H, *J*_H-H_ = 7 Hz); 6.87 (d, 2H, o-Ar-H, *J*_H-H_ = 8 Hz); 7.12 (d, 1H, H-8 of quinolone, *J*_H-F_ = 7 Hz); 7.17 (t, 2H, m-Ar-H, *J*_H-H_ = 8 Hz); 7.29–7.48 (m, 5H, Ar-H); 7.91 (d, 1H, H-5 of quinolone, *J*_H-F_ = 13 Hz); and 8.93 (s, 1H, H-2 of quinolone). ^13^C-NMR (100 MHz, DMSO-*d*_6_): δ 15.3, 42.2, 45.8, 46.6, 49.3, 50.3, 50.6, 51.1, 69.7, 107.0, 108.1, 112.2 (*J*_C-F_ = 21 Hz), 116.3, 119.7, 120.4, 128.8, 129.3, 129.8, 130.1, 136.9, 138.0, 145.9 (*J*_C-F_ = 10), 149.5, 151.9, 153.7 (*J*_C-F_ = 252 Hz), 167.0, 168.6 and 177.1.

#### 3.3.7. 1-Cyclopropyl-6-fluoro-7-(4-(2-(4-(2-methoxyphenyl)piperazin-1-yl)acetyl)piperazin-1-yl)-4-oxo-1,4-dihydroquinoline-3-carboxylic acid (**CD-5**)

Method A: Compound **CD-1** (0.5 g, 1.2 mmol); 2-methoxyphenylpiperazine as its HCl salt (0.55 g, 2.4 mmol) and triethylamine (0.67 mL, 4.8 mmol). **CD-5** was crystallized from acetonitrile to give 69% yield of off-white crystals; m.p. 242–245 °C; IR, cm^−1^, (KBr): 3100–3003 (Ar-H), 2929–2823 (–CH), 1726 (C=O, acid), 1647 (C=O, amide), 1629 (C=O, keto), 1498–1440 (C=C); LC-MS (APCI) *m/z*: 564 ([M+H]^+^, 100%); HRMS *m/z* calculated: 564.26222, found: 564.26167; ^1^H-NMR(400 MHz, DMSO-*d*_6_): δ 1.16–1.29 (m, 4H, cyclopropyl); 2.58 (bs, 4H, piperazine); 2.96 (bs, 4H, piperazine); 3.27 (s, 2H, COCH_2_–); 3.30–3.38 (bs, 4H, piperazine); 3.69 (bs, 2H, piperazine); 3.75 (s, 3H, OCH_3_); 3.80 (tt, 1H, cyclopropyl); 3.80 (bs, 4H, piperazine); 6.82–6.94 (m, 4H, Ar-H); 7.54–7.56 (d, 1H, H-8 of quinolone, *J*_H-F_ = 7 Hz); 7.86 (d, 1H, H-5 of quinolone, *J*_H-F_ = 13 Hz); and 8.63 (s, 1H, H-2 of quinolone). ^13^C-NMR (100 MHz, DMSO-*d*_6_): δ 8.0, 36.3, 41.3, 45.3, 49.8, 50.3, 50.5, 53.2, 55.7, 61.1, 107.0, 107.1, 111.4 (*J*_C-F_ = 22 Hz), 112.3, 118.3, 119.2, 121.2, 122.9, 139.5, 141.5, 145.4 (*J*_C-F_ = 12 Hz), 148.4, 152.4, 153.3 (*J*_C-F_ = 247 Hz) 166.3, 168.1 and 176.7.

#### 3.3.8. 1-Cyclopropyl-6-fluoro-7-(4-(2-(4-(2-methoxyphenyl)piperazin-1-yl)-2-phenylacetyl)piperazin-1-yl)-4-oxo-1,4-dihydroquinoline-3-carboxylic acid (**CD-6**)

Method B: Compound **CD-2** (0.5 g, 1.0 mmol); 2-methoxyphenylpiperazine as its HCl salt (0.46 g, 2 mmol) and triethylamine (0.56 mL, 4 mmol). **CD-6** was crystallized from ethyl acetate to give 35% yield of off-white crystals; m.p. 155–158 °C; IR, cm^−1^, (KBr): 3057–3000 (Ar-H), 2910–2825 (–CH), 1732 (C=O, acid), 1647 (C=O, amide), 1625 (C=O, keto), 1500–1435 (C=C); LC-MS (APCI) *m/z*: 640 ([M+H]^+^, 100%); HRMS *m/z* calculated: 640.29352, found: 640.29352; ^1^H-NMR(400 MHz, CDCl_3_): δ 1.17–1.36 (m, 4H, cyclopropyl); 2.83 (bs, 4H, piperazine); 3.15 (bs, 4H, piperazine); 3.43–3.51 (m, 4H, piperazine); 3.75–3.96 (m, 4H, piperazine); 3.75 (tt, 1H, cyclopropyl); 3.81 (s, 3H, OCH_3_); 4.48 (s, 1H, COCHPh); 6.81–6.99 (m, 4H, Ar-H); 7.17–7.48 (m, 5H, Ar-H); 7.33 (d, 1H, H-8 of quinolone, *J*_H-F_ = 7 Hz); 7.86 (d, 1H, H-5 of quinolone, *J*_H-F_ = 13 Hz); and 8.65 (s, 1H, H-2 of quinolone). ^13^C-NMR (100 MHz, CDCl_3_): δ 8.3, 35.5, 41.9, 45.3, 49.4, 49.8, 50.5, 51.7, 55.4, 71.9, 105.2, 108.0, 111.2, 112.4 (*J*_C-F_ = 24 Hz), 118.3, 120.0, 121.1, 123.2, 128.6, 129.0, 129.4, 134.7, 139.0, 141.0, 145.4 (*J*_C-F_ = 12 Hz), 147.6, 152.3, 153.3 (*J*_C-F_ = 290 Hz), 166.9, 169.1 and 177.0.

#### 3.3.9. 1-Ethyl-6-fluoro-7-(4-(2-(4-(2-methoxyphenyl)piperazin-1-yl)acetyl)piperazin-1-yl)-4-oxo-1,4-dihydroquinoline-3-carboxylic acid (**ND-5**)

Method A: Compound **ND-1** (0.5 g, 1.3 mmol); 2-methoxyphenylpiperazine as its HCl salt (0.59 g, 2.6 mmol) and triethylamine (0.72 mL, 5.2 mmol). **ND-5** was crystallized from acetonitrile to give 51% yield of off-white crystals; m.p. 244–247 °C; IR, cm^−1^, (KBr): 3039–2993 (Ar-H), 2939–2823 (–CH), 1716 (C=O, acid), 1654 (C=O, amide), 1625 (C=O, keto), 1514–1450 (C=C); LC-MS (APCI) *m/z*: 552 ([M+H]^+^, 100%); HRMS *m/z* calculated: 552.26222, found: 552.26167; ^1^H-NMR(400 MHz, DMSO-*d*_6_): δ 1.40 (t, 3H, CH_3_, *J*_H-H_ = 7 Hz); 2.57 (bs, 4H, piperazine); 2.95 (bs, 4H, piperazine); 3.26 (s, 2H, COCH_2_–); 3.30 (bs, 4H, piperazine); 3.67 (bs, 2H, piperazine); 3.75 (s, 3H, OCH_3_); 3.79 (bs, 2H, piperazine); 4.58 (q, 2H, CH_2_Me, *J*_H-H_ = 7 Hz); 6.83–6.91 (m, 4H, Ar-H); 7.18–7.20 (d, 1H, H-8 of quinolone, *J*_H-F_ = 7 Hz); 7.91 (d, 1H, H-5 of quinolone, *J*_H-F_ = 13 Hz); and 8.94 (s, 1H, H-2 of quinolone). ^13^C-NMR (100 MHz, DMSO-*d*_6_): δ 15.3, 41.5, 41.9, 45.9, 50.0, 50.4, 51.0, 53.7, 56.3, 61.7, 107.1, 108.1, 112.2 (*J*_C-F_ = 23), 112.8, 118.3, 120.4, 121.8, 123.4, 138.1, 142.1, 146.1 (*J*_C-F_ = 12 Hz), 149.5, 152.9, 153.7 (*J*_C-F_ = 241 Hz), 167.1, 168.6 and 177.1.

#### 3.3.10. 1-Ethyl-6-fluoro-7-(4-(2-(4-(2-methoxyphenyl)piperazin-1-yl)-2-phenylacetyl)piperazin-1-yl)-4-oxo-1,4-dihydroquinoline-3-carboxylic acid (**ND-6**)

Method B: Compound **ND-2** (0.5 g, 1.1 mmol); 2-methoxyphenylpiperazine as its HCl salt (0.50 g, 2.2 mmol) and triethylamine (0.61 mL, 4.4 mmol). **ND-6** was crystallized from ethyl acetate to give 42% yield of off-white crystals; m.p. 220–222 °C; IR, cm^−1^, (KBr): 3100–3010 (Ar-H), 2914–2831 (–CH), 1732 (C=O, acid), 1645 (C=O, amide), 1627 (C=O, keto), 1500–1446 (C=C); LC-MS (APCI) *m/z*: 628 ([M+H]^+^, 100%); HRMS *m/z* calculated: 628.29352, found: 628.29297; ^1^H-NMR(400 MHz, DMSO-*d*_6_): δ 1.37–1.40 (t, 3H, CH_3_, *J*_H-H_ = 7 Hz); 2.60 (bs, 4H, piperazine); 2.93 (bs, 4H, piperazine); 3.26 (bs, 4H, piperazine); 3.53–3.72 (m, 4H, piperazine); 3.63 (s, 3H, OCH_3_); 4.56 (q, 2H, CH_2_Me, *J*_H–H_ = 7 Hz); 4.67 (s, 2H, COCHPh); 6.84–6.89 (m, 4H, Ar-H); 7.13 (d, 1H, H-8 of quinolone, *J*_H-F_ = 7 Hz); 7.28–7.47 (m, 5H, Ar-H) 7.91 (d, 1H, H-5 of quinolone, *J*_H-F_ = 13 Hz); and 8.94 (s, 1H, H-2 of quinolone). ^13^C-NMR (100 MHz, DMSO-*d*_6_): δ 15.3, 41.5, 42.1, 45.8, 50.9, 50.2, 50.7, 51.3, 56.2, 69.6, 107.1, 108.1, 112.1 (*J*_C-F_ = 23 Hz), 112.8, 118.9, 120.4, 121.7, 123.3, 128.7, 129.3, 130.1, 136.9, 138.1, 142.2, 146.1 (*J*_C-F_ = 12 Hz), 149.5, 152.9, 153.7 (*J*_C-F_ = 240 Hz), 167.1, 168.7 and 177.1.

#### 3.3.11. 7-(4-(2-(4-(3-Chlorophenyl)piperazin-1-yl)acetyl)piperazin-1-yl)-1-cyclopropyl-6-fluoro-4-oxo-1,4-dihydroquinoline-3-carboxylic acid (**CD-7**)

Method A: Compound **CD-1** (0.5 g, 1.2 mmol); 3-chlorophenylpiperazine as its HCl salt (0.56 g, 2.4 mmol) and triethylamine (0.67 mL, 4.8 mmol). **CD-7** was crystallized from acetonitrile to give 40% yield of off-white crystals; m.p. 234–236 °C; IR, cm^−1^, (KBr): 3049–3010 (Ar-H), 2914–2819 (–CH), 1735 (C=O, acid), 1654 (C=O, amide), 1625 (C=O, keto), 1508–1458 (C=C); LC-MS (APCI) *m/z*: 568 ([M+H]^+^, 100%); HRMS *m/z* calculated: 568.21289, found: 568.21214; ^1^H-NMR (400 MHz, DMSO-*d*_6_): δ 1.16–1.28 (m, 4H, cyclopropyl); 2.57 (bs, 4H, piperazine); 3.17 (bs, 4H, piperazine); 3.28(s, 2H, COCH_2_–); 3.30–3.36 (bs, 4H, piperazine); 3.69 (bs, 2H, piperazine); 3.79 (tt, 1H, cyclopropyl); 3.79 (bs, 2H, piperazine); 6.75 (d, 1H, 6-Ar-H, *J*_H-H_ = 8 Hz); 6.87 (d, 1H, 4-Ar-H, *J*_H-H_ = 9 Hz); 6.91 (s, 1H, 2-Ar-H); 7.18 (t, 1H, 5-Ar-H, *J*_H-H_ = 8 Hz); 7.54 (d, 1H, H-8 of quinolone, *J*_H-F_ = 7 Hz); 7.88 (d, 1H, H-5 of quinolone, *J*_H-F_ = 13 Hz); and 8.62 (s, 1H, H-2 of quinolone). ^13^C-NMR (100 MHz, DMSO-*d*_6_): δ 8.0, 36.2, 41.3, 45.3, 48.1, 49.8, 50.3, 52.6, 60.8, 107.0, 107.1, 111.4 (*J*_C-F_ = 22 Hz), 114.1, 114.9, 118.5, 119.2, 130.8, 134.2, 139.5, 145.3 (*J*_C-F_ = 12 Hz), 148.4, 152.6, 153.3 (*J*_C-F_ = 241 Hz) 166.3, 167.9 and 176.7.

#### 3.3.12. 7-(4-(2-(4-(3-Chlorophenyl)piperazin-1-yl)-2-phenylacetyl)piperazin-1-yl)-1-cyclopropyl-6-fluoro-4-oxo-1,4-dihydroquinoline-3-carboxylic acid (**CD-8**)

Method B: Compound **CD-2** (0.5 g, 1.0 mmol); 3-chlorophenylpiperazine as its HCl salt (0.46 g, 2 mmol) and triethylamine (0.56 mL, 4 mmol). **CD-8** was crystallized from acetonitrile to give 60% yield of off-white crystals; m.p. 211–213 °C; IR, cm^−1^, (KBr): 3057–3045 (Ar-H), 2910–2237 (–CH), 1728 (C=O, acid), 1650 (C=O, amide), 1625 (C=O, keto), 1508–1456 (C=C); LC-MS (APCI) *m/z*: 644 ([M+H]^+^, 100%); HRMS *m/z* calculated: 644.24344, found: 644.43495; ^1^H-NMR(400 MHz, DMSO*-d*_6_): δ 1.13–1.28 (m, 4H, cyclopropyl); 2.56–2.57 (bs, 4H, piperazine); 3.14 (bs, 4H, piperazine); 3.23 (bs, 4H, piperazine); 3.67–3.91 (bs, 4H, piperazine); 3.73 (tt, 1H, cyclopropyl); 4.67 (s, 2H, COCHPh); 6.72–6.74 (d, 1H, 6-Ar-H, *J*_H-H_ = 8 Hz); 6.81–6.84 (d, 1H, 4-Ar-H, *J*_H-H_ = 9 Hz); 6.87 (s, 1H, 2-Ar-H); 7.14–7.18 (t, 1H, 5-Ar-H, *J*_H-H_ = 8 Hz); 7.29–7.39 (m, 5H, Ar-H); 7.45–7.47 (d, 1H, H-8 of quinolone, *J*_H-F_ = 7 Hz); 7.83–7.87 (d, 1H, H-5 of quinolone, *J*_H-F_ = 13 Hz); and 8.62 (s, 1H, H-2 of quinolone). ^13^C-NMR (100 MHz, CDCl_3_): δ 8.2, 35.3, 41.7, 45.1, 48.5, 49.2, 49.6, 50.9, 71.5, 105.0, 107.8, 112.1 (*J*_C-F_ = 23), 113.7, 115.5, 119.2, 119.8, 128.5, 128.9, 129.1, 130.0, 134.6, 134.8, 138.9, 145.2 (*J*_C-F_ = 10), 147.4, 152.1, 153.4 (*J*_C-F_ = 250), 166.7, 169.0 and 176.8.

#### 3.3.13. 7-(4-(2-(4-(3-Chlorophenyl)piperazin-1-yl)acetyl)piperazin-1-yl)-1-ethyl-6-fluoro-4-oxo-1,4-dihydroquinoline-3-carboxylic acid (**ND-7**)

Method A: Compound **ND-1** (0.5 g, 1.3 mmol); 3-chlorophenylpiperazine as its HCl salt (0.59 g, 2.6 mmol) and triethylamine (0.72 mL, 5.2 mmol). **ND-7** was crystallized from acetonitrile to give 38% yield of pale-yellow crystals; m.p. 217–220 °C; IR, cm^−1^, (KBr): 3100–3050 (Ar-H), 2900–2829 (–CH), 1720 (C=O, acid), 1653 (C=O, amide), 1627 (C=O, keto), 1516–1446 (C=C); LC-MS (APCI) *m/z*: 556 ([M+H]^+^, 40%); HRMS *m/z* calculated: 556.21269, found: 556.21214; ^1^H-NMR (400 MHz, DMSO-*d*_6_): δ 1.38 (t, 3H, CH_3_, *J*_H-H_ = 7 Hz); 2.56 (bs, 4H, piperazine); 3.16 (bs, 4H, piperazine); 3.26 (s, 2H, COCH_2_–); 3.29 (bs, 4H, piperazine); 3.67 (bs, 2H, piperazine); 3.78 (bs, 2H, piperazine); 4.54 (q, 2H, CH_2_Me, *J*_H-H_ = 7 Hz); 6.74 (d, 1H, 6-Ar-H, *J*_H-H_ = 8 Hz); 6.85 (d, 1H, 4-Ar-H, *J*_H-H_ = 8 Hz); 6.89 (s, 1H, 2-Ar-H); 7.14 (t, 1H, 5-Ar-H, *J*_H-H_ = 8 Hz); 7.19 (d, 1H, H-8 of quinolone, *J*_H-F_ = 7 Hz); 7.83 (d, 1H, H-5 of quinolone, *J*_H-F_ = 13 Hz); and 8.89 (s, 1H, H-2 of quinolone). ^13^C-NMR (100 MHz, DMSO-*d*_6_): δ 15.3, 41.9, 45.9, 48.6, 50.0, 50.3, 50.9, 53.2, 61.4, 106.9, 108.0, 112.1 (*J*_C-F_ = 22), 114.6, 115.5, 119.0, 120.3, 131.3, 134.7, 138.0, 146.1 (*J*_C-F_ = 9 Hz), 149.4, 153.1, 153.7 (*J*_C-F_ = 257 Hz), 167.0, 168.5 and 177.0.

#### 3.3.14. 7-(4-(2-(4-(3-Chlorophenyl)piperazin-1-yl)-2-phenylacetyl)piperazin-1-yl)-1-ethyl-6-fluoro-4-oxo-1,4-dihydroquinoline-3-carboxylic acid (**ND-8**)

Method B: Compound **ND-2** (0.5 g, 1.1 mmol); 3-chlorophenylpiperazine as its HCl salt (0.50 g, 2.2 mmol) and triethylamine (0.61 mL, 4.4 mmol). **ND-8** was crystallized from acetonitrile to give 58% yield of pale-yellow crystals; m.p. 186–189 °C; IR, cm^−1^, (KBr): 3050–3010 (Ar-H), 2910–2829 (–CH), 1716 (C=O, acid), 1650 (C=O, amide), 1625 (C=O, keto), 1514–1440 (C=C); LC-MS (APCI) *m/z*: 632 ([M+H]^+^, 20%); HRMS *m/z* calculated: 632.24399, found: 631.24344; ^1^H-NMR (400 MHz, DMSO*-d*_6_): δ 1.37 (t, 3H, CH_3_, *J*_H-H_ = 7 Hz); 2.59 (m, 4H, piperazine); 3.14 (bs, 4H, piperazine); 3.24 (bs, 4H, piperazine); 3.65–3.90 (m, 4H, piperazine); 4.54 (q, 2H, CH_2_Me, *J*_H-H_ = 7 Hz); 4.67 (s, 2H, COCHPh); 6.73 (d, 1H, 6-Ar-H, *J*_H-H_ = 8 Hz), 6.82 (d, 1H, 4-Ar-H, *J*_H-H_ = 9 Hz); 6.86 (s, 1H, 2-Ar-H); 7.09 (d, 1H, H-8 of quinolone, *J*_H-F_ = 7 Hz); 7.15 (t, 1H, 5-Ar-H, *J*_H-H_ = 8 Hz); 7.30–7.47 (m, 5H, Ar-H); 7.85 (d, 1H, H-5 of quinolone, *J*_H-F_ = 13 Hz); and 8.91 (s, 1H, H-2 of quinolone). ^13^C-NMR (100 MHz, DMSO-*d*_6_): δ 14.8, 41.6, 45.2, 48.2, 49.4, 49.7, 50.1, 50.25, 68.93, 106.43, 107.47, 111.58 (*J*_C-F_ = 23 Hz), 113.94, 114.79, 118.29, 119.85, 128.26, 128.77, 129.5, 130.8, 134.2, 136.3, 137.5, 145.4, 148.9 (*J*_C-F_ = 12 Hz), 152.6, 153.1 (*J*_C-F_ = 246 Hz), 166.5, 169.1 and 176.5.

#### 3.3.15. 1-Cyclopropyl-6-fluoro-4-oxo-7-(4-(2-(4-(pyrimidin-2-yl)piperazin-1-yl)acetyl)piperazin-1-yl)-1,4-dihydroquinoline-3-carboxylic acid (**CD-9**)

Method A: Compound **CD-1** (0.5 g, 1.2 mmol); pyrimidylpiperazine as its 2HCl salt (0.57 g, 2.4 mmol) and triethylamine (1.00 mL, 7.2 mmol). **CD-9** was crystallized from acetonitrile to give 25% yield of pale-yellow crystals; m.p. 219–221 °C; IR, cm^−1^, (KBr): 3100–3000 (Ar-H), 2920–2840 (–CH), 1716 (C=O, acid), 1650 (C=O, amide), 1629 (C=O, keto), 1500–1442 (C=C); LC-MS (APCI) *m/z*: 536 ([M+H]^+^, 100%); HRMS *m/z* calculated: 536.24216, found: 536.24161; ^1^H-NMR (400 MHz, DMSO-*d*_6_): δ 1.17–1.30 (m, 4H, cyclopropyl); 3.28 (s, 2H, COCH_2_–); 3.34 (bs, 8H, piperazine); 3.69 (tt, 1H, cyclopropyl); 3.73–3.88 (m, 8H, piperazine); 6.60 (t, 1H, pyrimidine, *J*_H-H_ = 5 Hz); 7.55 (d, 1H, H-8 of quinolone, *J*_H-F_ = 7 Hz); 7.88 (d, 1H, H-5 of quinolone, *J*_H-F_ = 13 Hz); 8.33 (d, 2H, pyrimidine, *J*_H-H_ = 5 Hz); and 8.63 (s, 1H, H-2 of quinolone). ^13^C-NMR (100 MHz, DMSO-*d*_6_): δ 8.0, 36.3, 41.3, 43.7, 453, 49.7, 50.2, 52.6, 60.8, 107.0, 107.1, 110.6, 111.4 (*J*_C-F_ = 23 Hz), 119.2, 139.5, 145.3 (*J*_C-F_ = 9 Hz), 148.4, 153.9 (*J*_C-F_ = 249 Hz), 158.3, 161.6, 166.3, 167.9 and 176.7.

#### 3.3.16. 1-Cyclopropyl-6-fluoro-4-oxo-7-(4-(2-phenyl-2-(4-(pyrimidin-2-yl)piperazin-1-yl)acetyl)piperazin-1-yl)-1,4-dihydroquinoline-3-carboxylic acid (**CD-10**)

Method B: Compound **CD-2** (0.5 g, 1.0 mmol); pyrimidylpiperazine as its 2HCl salt (0.47 g, 2 mmol) and triethylamine (0.83 mL, 6 mmol). **CD-10** was crystallized from ethyl acetate to give 16% yield of pale-yellow crystals; m.p. 202–205 °C; IR, cm^−1^, (KBr): 3050–3000 (Ar-H), 2900–2840 (–CH), 1725 (C=O, acid), 1650 (C=O, amide), 1627 (C=O, keto), 1506–1435 (C=C); LC-MS (APCI) *m/z*: 612 ([M+H]^+^, 100%); HRMS *m/z* calculated: 612.27346, found: 612.27291; ^1^H-NMR (400 MHz, DMSO*-d*_6_): δ 1.14–1.29 (m, 4H, cyclopropyl); 2.95–3.23 (m, 8H, piperazine); 3.57–3.89 (m, 8H, piperazine); 3.76 (tt, 1H, cyclopropyl); 4.62 (s, H, COCHPh); 6.60 (t, 1H, pyrimidine, *J*_H-H_ = 4 Hz); 7.35 (d, 1H, H-8 of quinolone, *J*_H-F_ = 7 Hz); 7.39–7.52 (m, 5H, Ar-H); 7.86 (d, 1H, H-5 of quinolone, *J*_H-F_ = 13 Hz); 8.33 (d, 2H, pyrimidine, *J*_H-H_ = 4 Hz); and 8.63 (s, 1H, H-2 of quinolone). ^13^C-NMR (100 MHz, DMSO-*d*_6_): δ 8.00, 36.3, 41.7, 45.2, 49.5, 49.8, 50.3, 57.8, 69.0, 106.9, 107.1, 110.6, 111.4 (*J*_C-F_ = 23 Hz), 119.2, 128.7, 128.9, 129.1, 129.6, 139.5, 145.0 (*J*_C-F_ = 6 Hz), 148.5, 153.3 (*J*_C-F_ = 248 Hz), 158.3, 161.5, 165.8, 166.3 and 176.7.

#### 3.3.17. 1-Ethyl-6-fluoro-4-oxo-7-(4-(2-(4-(pyrimidin-2-yl)piperazin-1-yl)acetyl)piperazin-1-yl)-1,4-dihydroquinoline-3-carboxylic acid (**ND-9**)

Method A: Compound **ND-1** (0.5 g, 1.3 mmol); pyrimidylpiperazine as its 2HCl salt (0.62 g, 2.6 mmol) and triethylamine (1.1 mL, 7.8 mmol). **ND-9** was crystallized from acetonitrile to give 51% yield of off-white crystals; m.p. 253–256 °C; IR, cm^−1^, (KBr): 3100–3010 (Ar-H), 2918–2820 (–CH), 1722 (C=O, acid), 1647 (C=O, amide), 1631 (C=O, keto), 1543–1442 (C=C); LC-MS (APCI) *m/z*: 524 ([M+H]^+^, 100%); HRMS *m/z* calculated: 524.24216, found: 524.24161; ^1^H-NMR (400 MHz, DMSO-*d*_6_): δ 1.38–1.42 (t, 3H, CH_3_, *J*_H-H_ = 7 Hz); 3.26 (s, 2H, COCH_2_–); 3.35 (bs, 8H, piperazine); 3.67–3.80 (m, 8H, piperazine); 4.57 (q, 2H, CH_2_Me, *J*_H-H_ = 7 Hz); 6.60 (t, 1H, pyrimidine, *J*_H-H_ = 5 Hz); 7.17 (d, 1H, H-8 of quinolone, *J*_H-F_ = 7 Hz); 7.89 (d, 1H, H-5 of quinolone, *J*_H-F_ = 13 Hz); 8.32 (d, 2H, pyrimidine, *J*_H-H_ = 5 Hz); and 8.92 (s, 1H, H-2 of quinolone). ^13^C-NMR (100 MHz, DMSO-*d*_6_): δ 15.3, 41.9, 44.3, 45.9, 50.0, 50.3, 50.9, 53.2, 61.4, 107.0, 108.0, 111.1, 112.2 (*J*_C-F_ = 22 Hz), 120.4, 138.1, 146.2 (*J*_C-F_ = 9 Hz), 149.5, 153.8 (*J*_C-F_ = 243 Hz), 158.9, 162.1, 167.0, 168.5 and 177.1.

#### 3.3.18. 1-Ethyl-6-fluoro-4-oxo-7-(4-(2-phenyl-2-(4-(pyrimidin-2-yl)piperazin-1-yl)acetyl)piperazin-1-yl)-1,4-dihydroquinoline-3-carboxylic acid (**ND-10**)

Method B: Compound **ND-2** (0.5 g, 1.1 mmol); pyrimidylpiperazine as its 2HCl salt (0.52 g, 2.2 mmol) and triethylamine (0.92 mL, 6.6 mmol). **ND-10** was crystallized from acetonitrile to give 43% yield of pale-yellow crystals; m.p. 153–156 °C; IR, cm^−1^, (KBr): 3050–3000 (Ar-H), 2910–2810 (–CH), 1718 (C=O, acid), 1650 (C=O, amide), 1627 (C=O, keto), 1544–1446 (C=C); LC-MS (APCI) *m/z*: 522 ([M−Ph]^+^, 100%); HRMS *m/z* calculated: 600.27346, found: 600.27291; ^1^H-NMR (400 MHz, DMSO*-d*_6_): δ 1.37 (t, 3H, CH_3_, *J*_H-H_ = 7 Hz); 3.00–3.30 (m, 8H, piperazine); 3.55–3.80 (m, 8H, piperazine); 4.54 (q, 2H, CH_2_Me, *J*_H-H_ = 7 Hz); 4.68 (s, 1H, COCHPh); 6.57 (t, 1H, pyrimidine, *J*_H-H_ = 4 Hz); 7.08 (d, 1H, H-8 of quinolone, *J*_H-F_ = 7 Hz); 7.30–7.46 (m, 5H, Ar-H); 7.86 (d, 1H, H-5 of quinolone, *J*_H-F_ = 13 Hz); 8.30 (d, 2H, pyrimidine, *J*_H-H_ = 4 Hz); and 8.91 (s, 1H, H-2 of quinolone). ^13^C-NMR (100 MHz, DMSO*-d*_6_): δ 14.8, 41.6, 43.9, 45.2, 49.4, 49.7, 50.0, 50.3, 68.9, 106.4, 107.5, 110.4, 111.5 (*J*_C-F_ = 23 Hz), 119.8, 128.3, 128.8, 129.6, 136.3, 137.5, 145.5 (*J*_C-F_ = 10 Hz), 148.9, 153.2 (*J*_C-F_ = 257 Hz), 158.3, 161.5, 166.5, 169.1 and 176.5.

#### 3.3.19. 7-(4-(2-(4-Benzylpiperidin-1-yl)acetyl)piperazin-1-yl)-1-cyclopropyl-6-fluoro-4-oxo-1,4-dihydroquinoline-3-carboxylic acid (**CD-11**)

Method A: Compound **CD-1** (0.5 g, 1.2 mmol); benzylpiperidine (0.43 mL, 2.4 mmol) and triethylamine (0.33 mL, 2.4 mmol). **CD-11** was crystallized from ethyl acetate/ether to give 28% yield of off-white crystals; m.p. 144–147 °C; IR, cm^−1^, (KBr): 3100–3010 (Ar-H), 2922–2810 (–CH), 1722 (C=O, acid), 1650 (C=O, amide), 1629 (C=O, keto), 1489–1442 (C=C); LC-MS (APCI) *m/z*: 547 ([M+H]^+^, 10%); HRMS *m/z* calculated: 547.27206, found: 547.27151; ^1^H-NMR (400 MHz, CDCl_3_): δ 1.22 (m, 2H, cyclopropyl); 1.37 (m, 2H, pyridine –CCH_2_C–); 1.40 (m, 2H, cyclopropyl); 1.57 (bs, 1H, pyridine–CH–); 1.68 (d, 2H, pyridine–CH_2_N–, *J*_H-H_ = 12 Hz); 2.20 (bs, 2H, pyridine–CH_2_N–); 2.53 (d, 2H, pyridine –CH_2_N–, *J*_H–H_ = 7 Hz); 2.94 (d, 2H, CH_2_–Ph, *J*_H-H_ = 10 Hz); 3.34 (m, 4H, piperazine); 3.31 (s, 2H, COCH_2_–); 3.57 (tt, 1H, cyclopropyl); 3.87 (bs, 4H, piperazine); 7.12 (d, 2H, o-Ar-H, * J*_H-H_ = 7 Hz); 7.18 (t, 1H, p-Ar-H, *J*_H-H_ = 7 Hz); 7.27 (t, 2H, m-Ar-H, *J*_H-H_ = 7 Hz); 7.35 (d, 1H, H-5 of quinolone, *J*_H-F_ = 7 Hz); 7.96 (d, 1H, H-8 of quinolone, *J*_H-F_ = 13 Hz); and 8.70 (s, 1H, H-2 of quinolone). ^13^C-NMR (100 MHz, CDCl_3_): δ 8.2, 31.9, 35.3, 37.2, 41.3, 42.9, 45.6, 49.4, 50.5, 53.6, 61.3, 105.1, 108.0, 112.4 (*J*_C-F_ = 23 Hz), 120.0, 125.9, 128.2, 129.0, 139.0, 140.3, 145.4 (*J*_C-F_ = 10 Hz), 147.5, 153.5 (*J*_C-F_ = 250 Hz), 166.8, 168.0 and 176.9.

#### 3.3.20. 7-(4-(2-(4-Benzylpiperidin-1-yl)acetyl)piperazin-1-yl)-1-ethyl-6-fluoro-4-oxo-1,4-dihydroquinoline-3-carboxylic acid (**ND-11**)

Method A: Compound **ND-1** (0.5 g, 1.3 mmol); benzylpiperidine (0.46 mL, 2.6 mmol) and triethylamine (0.36 mL, 2.6 mmol). **ND-11** was crystallized from ethyl acetate/ether to give 22% yield of pale-yellow crystals; m.p. 118–120 °C; IR, cm^−1^, (KBr): 3,080–3,010 (Ar-H), 2,926–2,846 (–CH), 1724 (C=O, acid), 1656 (C=O, amide), 1625 (C=O, keto), 1508–1446 (C=C); LC-MS (APCI) *m/z*: 535 ([M+H]^+^, 100%); HRMS *m/z* calculated: 535.27206, found: 535.27151; ^1^H-NMR (400 MHz, CDCl_3_): δ 1.45–1.47 (m, 2H, pyridine–CCH_2_C–); 1.61 (t, 2H, –CH_3_, *J*_H-H_ = 7 Hz); 1.60 (bs, 1H, pyridine–CH–); 1.71 (d, 2H, pyridine–CH_2_N–, *J*_H-H_ = 12 Hz); 2.44 (bs, 2H, pyridine-CH_2_N–); 2.55 (d, 2H, pyridine–CH_2_N–, *J*_H-H_ = 7 Hz); 3.04 (d, 2H, CH_2_-Ph); 3.29 (s, 2H, COCH_2_–); 3.38–3.49 (bs, 4H, piperazine); 3.85 (bs, 4H, piperazine); 4.36 (q, 2H, CH_2_Me, *J*_H-H_ = 7 Hz); 6.87 (d, 1H, H-5 of quinolone, *J*_H-F_ = 7 Hz); 7.12 (d, 2H, o-Ar-H, *J*_H-H_ = 7 Hz); 7.19 (t, 1H, p-Ar-H, *J*_H-H_ = 7 Hz); 7.28 (t, 2H, m-Ar-H, *J*_H-H_ = 7 Hz); 8.06 (d, 1H, H-8 of quinolone, *J*_H-F_ = 13 Hz ); and 8.71 (s, 1H, H-2 of quinolone). ^13^C-NMR (100 MHz, CDCl_3_): δ 14.5, 31.5, 36.9, 41.4, 42.7, 45.3, 49.5, 49.7, 50.4, 53.6, 61.3, 104.2, 108.4, 112.9 (*J*_C-F_ = 23 Hz), 121.0, 126.0, 128.2, 129.0, 137.0, 140.0, 145.6 (*J*_C-F_ = 11 Hz), 147.3, 153.4 (*J*_C-F_ = 250 Hz), 167.4, 167.8 and 176.9.

#### 3.3.21. 1-Cyclopropyl-7-(4-(2-(dibenzylamino)acetyl)piperazin-1-yl)-6-fluoro-4-oxo-1,4-dihydroquinoline-3-carboxylic acid (**CD-12**)

Method A: Compound **CD-1** (0.5 g, 1.2 mmol); dibenzylamine (0.46 mL, 2.4 mmol) and triethylamine (0.33 mL, 2.4 mmol). **CD-12** was crystallized from acetonitrile to give 40% yield of pale-yellow crystals; m.p. 179–181 °C; IR, cm^−1^, (KBr): 3053–3026 (Ar-H), 2908–2831 (–CH), 1734 (C=O, acid), 1647 (C=O, amide), 1625 (C=O, keto), 1508–1465 (C=C); LC-MS (APCI) *m/z*: 569 ([M+H]^+^, 30%); HRMS *m/z* calculated: 569.25641, found: 569.25586; ^1^H-NMR (400 MHz, DMSO*-d*_6_): δ 1.17–1.31 (m, 4H, cyclopropyl); 3.22 (bs, 4H, piperazine); 3.28 (s, 2H, COCH_2_–); 3.52 (bs, 2H, piperazine); 3.64 (bs, 2H, piperazine); 3.66 (s, 4H, CH_2_-Ph); 3.79 (tt, 1H, cyclopropyl); 7.25–7.34 (m, 10H, Ar-H); 7.51 (d, 1H, H-8 of quinolone, *J*_H-F_ = 7 Hz); 7.86 (d, 1H, H-5 of quinolone, *J*_H-F_ = 13 Hz); and 8.63 (s, 1H, H-2 of quinolone). ^13^C-NMR (100 MHz, DMSO-*d*_6_): δ 8.0, 36.2, 41.1, 44.7, 49.5, 50.0, 55.7, 57.8, 106.9, 107.1, 111.3 (*J*_C-F_ = 23 Hz), 119.1, 127.5, 128.6, 129.4, 138.8, 139.4, 145.2 (*J*_C-F_ = 11 Hz), 148.4, 153.3 (*J*_C-F_ = 247 Hz), 166.2, 168.8 and 176.6.

#### 3.3.22. 7-(4-(2-(Dibenzylamino)acetyl)piperazin-1-yl)-1-ethyl-6-fluoro-4-oxo-1,4-dihydroquinoline-3-carboxylic acid (**ND-12**)

Method A: Compound **ND-1** (0.5 g, 1.3 mmol); dibenzylamine (0.50 mL, 2.6 mmol) and triethylamine (0.36 mL, 2.6 mmol). **ND-12** was crystallized from acetonitrile to give 29% yield of pale-yellow crystals; m.p. 112–115 °C; IR, cm^−1^, (KBr): 3059–3026 (Ar-H), 2914–2833 (–CH), 1732 (C=O, acid), 1,650 (C=O, amide), 1627 (C=O, keto), 1514–1452 (C=C); LC-MS (APCI) *m/z*: 557 ([M+H]^+^, 100%); HRMS *m/z* calculated: 557.25641, found: 557.25586; ^1^H-NMR (400 MHz, DMSO-*d*_6_): δ 1.40 (t, 4H, CH_3_, *J*_H-H_ = 7 Hz); 3.21 (bs, 4H, piperazine); 3.28 (s, 2H, COCH_2_–); 3.51 (bs, 2H, piperazine); 3.59 (bs, 2H, piperazine); 3.63 (s, 4H, CH_2_-Ph); 4.55 (q, 2H, CH_2_Me, * J*_H-H_ = 7 Hz); 7.11 (d, 1H, H-8 of quinolone, *J*_H-F_ = 7 Hz); 7.24–7.34 (m, 10H, Ar-H); 7.85 (d, 1H, H-5 of quinolone, *J*_H–F_ = 13 Hz); and 8.91 (s, 1H, H-2 of quinolone). ^13^C-NMR (100 MHz, DMSO-*d*_6_): δ 15.3, 41.6, 45.3, 50.0, 50.1, 50.7, 56.1, 58.3, 107.0, 108.0, 112.1 (*J*_C-F_ = 22 Hz), 120.4, 128.1, 129.2, 130.0, 138.0, 139.3, 146.1 (*J*_C-F_ = 11 Hz), 149.4, 153.7 (*J*_C-F_ = 247 Hz), 167.0, 169.3 and 177.0.

### 3.4. In Vitro Antibacterial Activity Assays

MICs (μM) of the synthesized compounds were determined using the broth microdilution method in a 96-well microtiter plate (Cellstar^®^, Greiner Bio-One, Frickenhausen, Germany). The stock solution of each compound was prepared in DMSO under aseptic conditions. Mueller-Hinton broth containing 0.1% Tween 20 was prepared in order to be used in the experiment. The first experimental well was filled with Mueller-Hinton broth (190 μL), and the other wells were filled with 100 μL of the same broth. A volume of 10 μL of each substances’ stock solutions was added to the first well. Double fold serial dilution was carried out across the plate. Overnight batch culture for standard microorganisms, *Pseudomonas aeruginosa* ATCC 9027, *Escherichia coli* ATCC 8739, *Staphylococcus aureus* ATCC 6538P and *Bacillus subtilis* ATCC 6633 (10 μL of each culture) was used to inoculate the wells, so as to achieve a final inoculum size of 5 × 10^5^ CFU/mL. The plate was incubated for 24 h at 37 °C. MIC was expressed as the mean of molar concentration between the first wells showing no growth. Growth was detected as turbidity (360 nm) relative to an un-inoculated well using microtiter plate reader (Biotek, Winooski, VT, USA). Negative controls were performed with sterile broth only in each well, and positive controls were performed with overnight culture only and 10 μL DMSO in each well. Each MIC determination was carried out in triplicate.

### 3.5. Quantitative Structure-Activity Relationships (QSAR)

PLS models were created using protocols built in Discovery Studio v3.0 by Accelrys (San Diego, CA, USA). Twenty four compounds were divided into two sets according to their parent compounds, *i.e.*, CIPRO derivatives (**IMD-1**, **CD-2**, **CD-3**, **CD-4**, **CD-5**, **CD-6**, **CD-7**, **CD-8**, **CD-9**, **CD-10**, **CD-11** and **CD-12**) and NOR derivatives (**IMD-2**, **ND-2**, **ND-3**, **ND-4**, **ND-5**, **ND-6**, **ND-7**, **ND-8**, **ND-9**, **ND-10**, **ND-11** and **ND-12**). PLS is a sequential algorithm that starts with an empty group and then adds one variable at a time to produce multiple prediction models, and the best model will be chosen by cross-validation. Two PLS models were created for each group, one for their antibacterial activity against *S. aureus* and the other for their activity against *B. subtilis*, to obtain four final models. In each model, cLog D, the molecular fractional polar surface area (FPSA) and Δ_C-13_ were the independent descriptors, while the antibacterial activity expressed as the log (1/MIC in µM) was the dependent variable. The “calculate molecular properties” protocol was used to calculate cLog D and the molecular fractional polar surface area (FPSA). Δ_C-13_ is the difference in ^13^C-NMR chemical shifts (δ) between the peak corresponding to carbonyl carbons of the amide and the carboxylic acid groups, δ_amide_ − δ_acid_, was obtained from mNova v7 by MestreLab (Santiago de Compostela, Spain) and was introduced into Discovery Studio manually. The models were validated using the leave-one-out cross-validation to evaluate how well the model will reproduce the data being analyzed and the prediction power of each model.

## 4. Conclusions

In this work, twenty-six derivatives of CIPRO and NOR were synthesized, and their activity was assayed against four different bacteria. The synthesized compounds, as intended, showed selectivity against Gram-positive bacteria, namely *S. aureus* and *B. subtilis.* In addition, the CIPRO derivatives were generally more potent than the NOR derivatives. While simple SAR deductions were informative, but not conclusive, QSAR computations showed that polarity, lipophilicity and electron density play a balanced role in the activity against *S. aureus*, while only electron density and lipophilicity seem to be important for the activity against *B. subtilis*. It is also worth mentioning that Compound **CD-10** is a good candidate for further investigation. It will be interesting to see the activity of this compound against other Gram-positive bacteria, and if it shows promise, enantiomeric separation or stereoselective synthesis of its two enantiomers in addition to an investigation of its molecular mechanism of action will be warranted. Although not the aim of this work, antibacterial activity against MRSA might be evaluated to obtain a more comprehensive profile of such compounds.
